# Yellow Fever

**Published:** 1884-08

**Authors:** 


### YELLOW FEVER.

DR. Dominigos Freire, appointed by the Brazilian Government to investigate the nature and cause of yellow fever outbreaks, has reported some of his researches. He has found in the soil of cemeteries where yellow fever subjects have been interred myriads of microbes, identical with those seen in the vomit, blood and urine of patients suffering from the fever, as well as vibriones in rapid motion. Dr. Freire believes that, after passing through the porosities of the earth, these germs disperse themselves in the atmosphere, while others are carried by storm rains to the towns, and there provoke epidemics of the disease. He proposes that the bodies of all persons who die of yellow fever be cremated.

